# Homozygous Missense Variant in the Solute Carrier Organic Anion Transporter 2A1 (*SLCO2A1*) Gene Underlies Isolated Nail Clubbing

**DOI:** 10.3390/genes14020430

**Published:** 2023-02-08

**Authors:** Muhammad Umair, Muhammad Bilal, Khadim Shah, Gulab Said, Farooq Ahmad

**Affiliations:** 1 Medical Genomics Research Department, King Abdullah International Medical Research Center (KAIMRC), Ministry of National Guard Health Affairs (MNGH), King Saud Bin Abdulaziz University for Health Sciences, Riyadh 14611, Saudi Arabia; 2 Department of Life Sciences, School of Science, University of Management and Technology (UMT), Lahore 54770, Punjab, Pakistan; 3 Department of Biochemistry, Faculty of Biological Sciences, Quaid-i-Azam University, Islamabad 45320, Pakistan; 4 Department of Dermatology, Yale School of Medicine, Yale University, New Heaven, CT 06511, USA; 5 Department of Chemistry, Women University Swabi, Swabi 23430, Khyber Pakhtunkhwa (KPK), Pakistan; 6 Department of Biochemistry, Women University Swabi, Swabi 23430, Khyber Pakhtunkhwa (KPK), Pakistan

**Keywords:** isolated congenital nail clubbing, novel missense variant, *SLCO2A1* gene

## Abstract

Background: Inherited isolated nail clubbing is a very rare Mendelian condition in humans, characterized by enlargement of the terminal segments of fingers and toes with thickened nails. Mutations in two genes have been reported to cause isolated nail clubbing in humans, which are the *SLCO2A1* gene and the *HPGD* gene. Objectives: An extended Pakistani family having two affected siblings born of unaffected consanguineous union was included in the study. Predominant isolated congenital nail clubbing (ICNC) without any other systemic abnormalities was observed, which we aimed to characterize at clinico-genetic level. Methods: Whole exome coupled with Sanger sequencing were employed to uncover the sequence variant as a cause of the disease. Furthermore, protein modeling was carried out to reveal the predicted possible effect of the mutation at the protein level. Results: Whole exome sequencing data analysis revealed a novel biallelic sequence variant (c.155T>A; p.Phe52Tyr) in the *SLCO2A1* gene. Further, Sanger sequencing analysis validated and confirmed the segregation of the novel variant in the entire family. Subsequently, protein modeling of the wild-type and mutated SLCO2A1 revealed broad-scale change, which might compromise the proteins’ secondary structure and function. Conclusion: The present study adds another mutation to the *SLCO2A1*-related pathophysiology. The involvement of *SLCO2A1* in the pathogenesis of ICNC may open exciting perceptions of this gene in nail development/morphogenesis.

## 1. Introduction

Isolated congenital nail clubbing (ICNC; OMIM 119900) is a rare genetic nail abnormality characterized by bilateral, symmetric bulbous enlargement of the distal segments of the finger- and/or toenail plate [1,2]. It results from unusual proliferation of the connective tissues between the distal phalanx and the nail matrix, and is associated with a hypoplastic, thick-ended, long, broad nail. The thumbs are almost always involved, although some fingers or toes may be spared. Clubbed fingers/nails are also known as drumstick fingers, watch-glass nails, and Hippocratic fingers/nails [3]. ICNC has been associated with various clinical conditions, including congenital heart disease, lung cancer, bronchiectasis, cirrhosis of the liver, etc. Digital clubbing is usually asymptomatic; however, it often reflects the presence of internal illness such as pulmonary fibrosis, lung cancer, or underlying addition severe conditions [3,4,5,6].

Genetic nail clubbing is either inherited in an autosomal dominant or recessive fashion with capricious penetrance. It may occur as a part of complex syndromes, which may include primary hypertrophic osteoarthropathy (PHOAR) and primary hypertrophic osteoarthropathy type 2 (PHOAR2) [1,2]. Primary hypertrophic osteoarthropathy (PHO) is a rare inherited disorder with variable expressivity predominantly manifested by clubbed shape of the fingernails and/or toenails, periostosis associated with bone formation of long bones, and arthropathy. Additionally, other associated features include pachydermia with facial roughening, thickened skin, seborrheic hyperplasia, and excess perspiration. Patients at early age have fontanels and delayed cranial suture closures.

Individuals affected with PHO showed 25% of persistent ductus arteriosus, whereas primary hypertrophic osteoarthropathy-2 (PHOAR2) is an autosomal recessive rare genetic abnormally presenting with deviation of normal to clubbed shaped digits, pachydermia, and periostosis. Patients with PHOAR2 often have painful ankles and knees accompanied by inflammation, watery diarrhea, and excessive hyperhidrosis. It has been observed that males are more severely affected by the disease as compared to females [1,2].

The predominant hallmark associated with primary hypertrophic osteoarthropathy (PHOAR) and primary hypertrophic osteoarthropathy type 2 (PHOAR2 is PGE2 (prostaglandin E 2) degradation was observed. A number of genes have been associated with transport and metabolism, including *HPGD* (*MIM 601688*), *PTGS1* (*MIM 176805*), *PTGS2* (*MIM 600262*), *PTGES* (*MIM 605172*), *PTGES2* (*MIM 608152*), *PTGES3* (*MIM 607061*), *PTGER1* (*MIM 176802*), *PTGER2* (*MIM 176804*), *PTGER3* (*MIM 176806*), *PTGER4* (*MIM 601586*)*, SLCO2A1* (*MIM 601460*), *SLCO3A1* (*MIM 612435*), *SLCO4A1* (*MIM 612436*), *PTGR1* (*MIM 601274*), and *PTGR2* (*MIM 608642*) [7]. However, only *SLCO2A1* (prostaglandin transporter) and *HPGD* encode the prostaglandin degrading enzyme 15-PGDH (15-hydroxyprostaglandin dehydrogenase) [8,9].

Here, we carried out clinical and genetic analysis of a family from Pakistan presenting autosomal recessive isolated nail clubbing, employing whole exome sequencing (WES) coupled with Sanger sequencing. WES data analysis led to the identification of a novel biallelic missense sequence variant in the *SLCO2A1* gene. Subsequent Sanger sequencing data analysis validated and confirmed the segregating of the variant within the family as the underlying cause of isolated nail clubbing.

## 2. Materials and Methods

### 2.1. Approval and DNA Extraction

**Figure 1:**
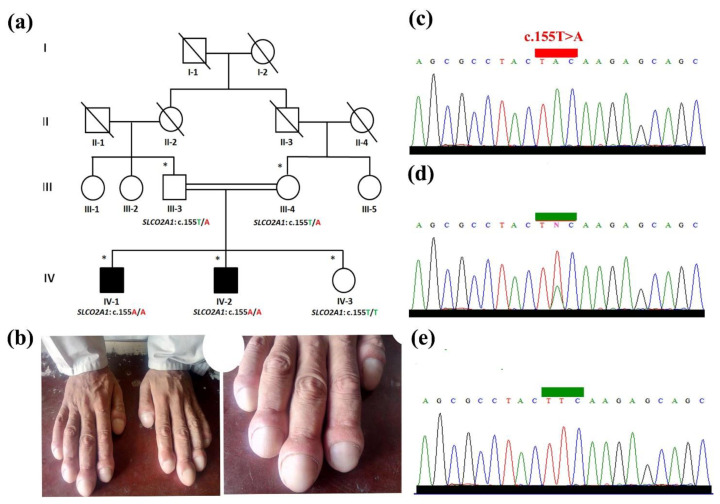
(**a**): Pedigree construction of the present family segregating isolated nail clubbing in an autosomal recessive fashion. The individuals included in the present study are represented by asterisks (*). (**b**) Hands of the proband (IV-1), presenting features of isolated nail clubbing. (**c**–**e**) Sanger sequencing analysis of the missense variant (c.155T>A) identified in the affected members and segregation in other family members.

The study was carried out according to the UMT institutional review board and Helsinki declaration. The family under study resided in the North Waziristan, Khyber Pakhtunkhwa (KP) province of the country, including two affected individuals with ICNC, inherited in an apparent autosomal recessive pattern. On the basis of detailed clinical information provided by the elders and the guardians, pedigree was drawn. The family was briefed about the study’s purpose, and for the smooth conduct of the study, necessary information including informed consent and clinical picture of the affected individuals was collected. Venus blood samples were collected from the two affected (IV-1, IV-2) and three unaffected (III-3, III-4, and IV-3) individuals (Figure 1a). For the genomic DNA extraction, purification and quantification was performed using standard methods described previously [10].

### 2.2. Whole Exome Sequencing (WES)

WES was carried out on the DNA sample of the affected members (IV-2) of the family. For this purpose, the Ion S5™ Sequencer was used, and sequence libraries were generated with the help of Ion AmpliSeq™ Exome RDY Kits. Step-by-step manufacturer protocols were followed as described previously [11,12], which included the following main steps: (a) amplification of the target DNA, (b) partial digestion of amplicons, (c) adaptor ligation and purification of amplicons, (d) DNA quantification and loading to Ion Chef™, (e) initialization and sequencing, and (f) filtration of variant data analysis.

### 2.3. Validation and Segregation Analysis by Sanger Sequencing

Employing various filtration steps, the WES data were carefully searched and analyzed for potential pathogenic variant/s, which were then Sanger sequenced for validation and segregation analysis in the entire family. The reference gene sequences along with exons, introns, 5′ and 3′ untranslated regions were obtained from the UCSC genome database browser. Primer sequences were generated bordering the variant/s of interest using the primer-3 software. The variant/s of interest were checked in the entire family including those of the affected, unaffected, and parents using Sanger sequencing following the established protocol [13]. Sanger sequencing results were analyzed using standard methods. Conservation of the mutated amino acid was identified using HomoloGene (NCBI, https://www.ncbi.nlm.nih.gov/homologene/).

### 2.4. In Silico Tools

In silico tools Mutation Taster, SIFT, Ployphen2, and CADD were used to check the possible pathogenic nature of the variants. The identified variants were also checked in different databases such as dbSNP, the 1000 Genome project, ExAC, gnomAD, the YH database, the HapMap Project, and our in-house exome dataset.

### 2.5. 3D Protein Modeling

To evaluate the effect at protein level, two different models of protein were created as a result of variants, one for the wild-type and the other for the mutated-type SLCO2A1 using standard methods. The UniProtKB protein databank was searched for the reference sequence of the SLCO2A1 and was found under as UniProtKB identifier ID: Q92959. After retrieving the reference sequence, it was processed for protein modeling through the available online server I-TASSER. A mutated version of the protein SLCO2A1PHE52TYR was created from the wild-type SLCO2A1WT through MODELLER {9.2114} [14,15].

## 3. Results

### 3.1. Clinical Evaluation

Both the affected individuals (IV-1, IV-2) (Figure 1a) were moved to the nearest government hospital for clinical evaluation and generation of extensive clinical profiles. During clinical evaluation, various aspects of the affected individuals were considered including age, weight, and height which were 28 years, 85 kg and 175 cm in the case of affected individual (IV-1), and 31 years, 82 kg, 173 cm and 24.8 BMI in the case of affected member (IV-2). The upper limbs of the patients were bilaterally affected by nail clubbing. Nails were characterized as broad, thick, and convex-shaped with shiny appearance. The nails were wider in diameter and had springy sensation on palpation. The nail blades were swelled up and hypoplastically broad (Figure 1b). The affected members were devoid of any unusual dermatological and hypohidrotic signs and symptoms. Physically and mentally they were normal with normal muscles, and without any joint pain. Detailed examination of the ECG (electrocardiography) by the cardiologist showed no significant change. Biochemical tests were unremarkable (serum PGE2 (117 pg/mL) urinary PGE2 (131 pg/mL) (normal: 25–200 pg/mL). They had no history of inflammatory bowel disease or graves disorder, mental illness, colon neoplasia, secondary hypertrophic osteoarthropathy, or any other type of abnormality. Detailed clinical evaluation of the entire family is shown in Table 1. Parents of the patients were clinically and genetically heterozygous unaffected, having normal nails on both fingers/toes and were phenotypically indistinguishable from other normal individuals.

### 3.2. Whole Exome Data Analysis

DNA of the affected members (IV-2) was selected and processed for the WES as described earlier [16]. Various step-by-step filtration inputs were employed for the WES data analysis, which led to the identification of a novel homozygous missense variant c.155T>A in the *SLCO2A1* gene (NM_005630.2) predicting the substitution of p. (Phe52Tyr) on chromosome 3q22.1-q22.2. The variant affects the nucleotide sequence of the exon 2 of *SLCO2A1* gene. To date, only one homozygous and one heterozygous variant have been identified in the *SLCO2A1* gene causing ICNC.

### 3.3. Validation and Segregation Analysis

**Figure 2:**
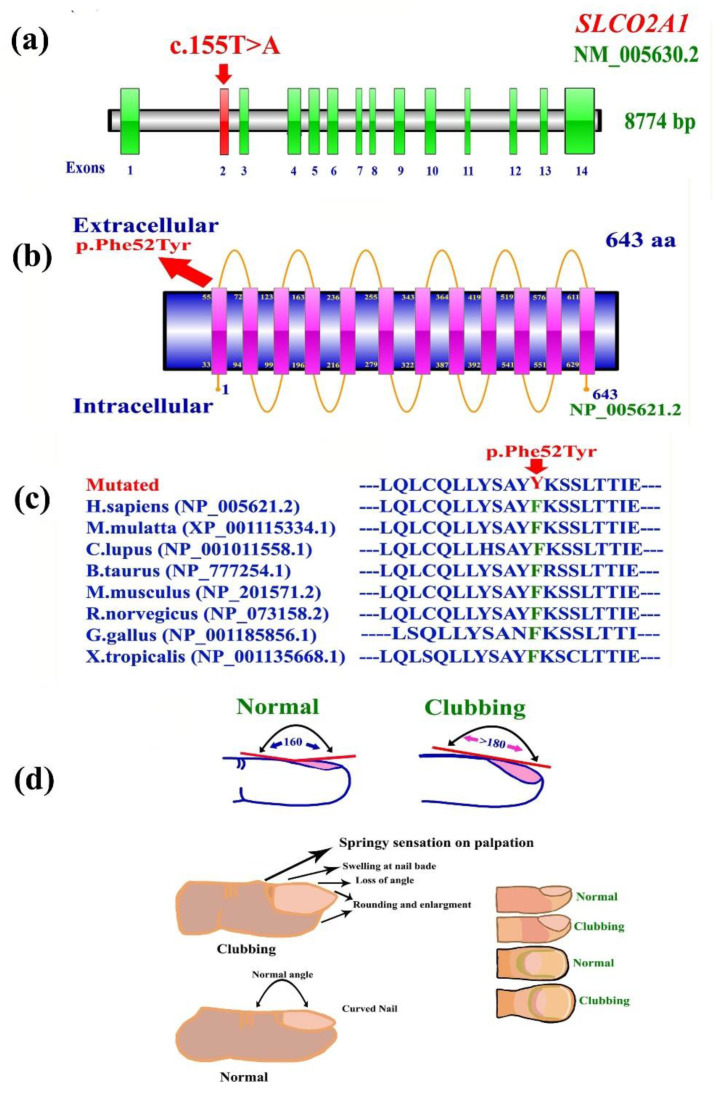
(**a**) *SLCO2A1* gene showing 14 coding exons, and position of mutation identified in the present study. (**b**) Cartoon model of prostaglandin transporter (SLCO2A1, PGT), position of identified mutation. PGT is an integral membrane protein with 12 transmembrane domains. Homozygous missense mutation Phe52Tyr is located within the first transmembrane domain of the protein. (**c**) Phe52 amino acid conservation across different species. (**d**) Cartoon representation of normal nail and nail clubbing.

The WES-identified novel missense sequence (c.155T>A; p.Phe52Tyr) in the *SLCO2A1* gene was subjected to validation and segregation analysis. For this purpose, PCR-based bi-directional Sanger sequencing was carried out on the entire family under study. Data analysis confirmed the segregation and validation of the novel pathogenic missense variant (c.155T>A; p.Phe52Tyr) with the disease phenotype (Figure 1c–e). The absence of the variant (c.155T>A) in ExAC, gnomAD, 1000 Genomes, and in-house exomes reveals the rare nature of the variant and will have a possible predicted pathogenic effect with a CADD phred score of 28 using CADD GRCh38-v1.6 and REVEL score of 0.8. The variant (c.155T>A) causes the change of phenylalanine by tyrosine at amino acid position 52. The variant Phe52Tyr is located in a highly conserved region that is conserved across different species (Figure 2c).

### 3.4. 3D Protein Modeling of SLCO2A1 (Phe52Tyr)

**Figure 3:**
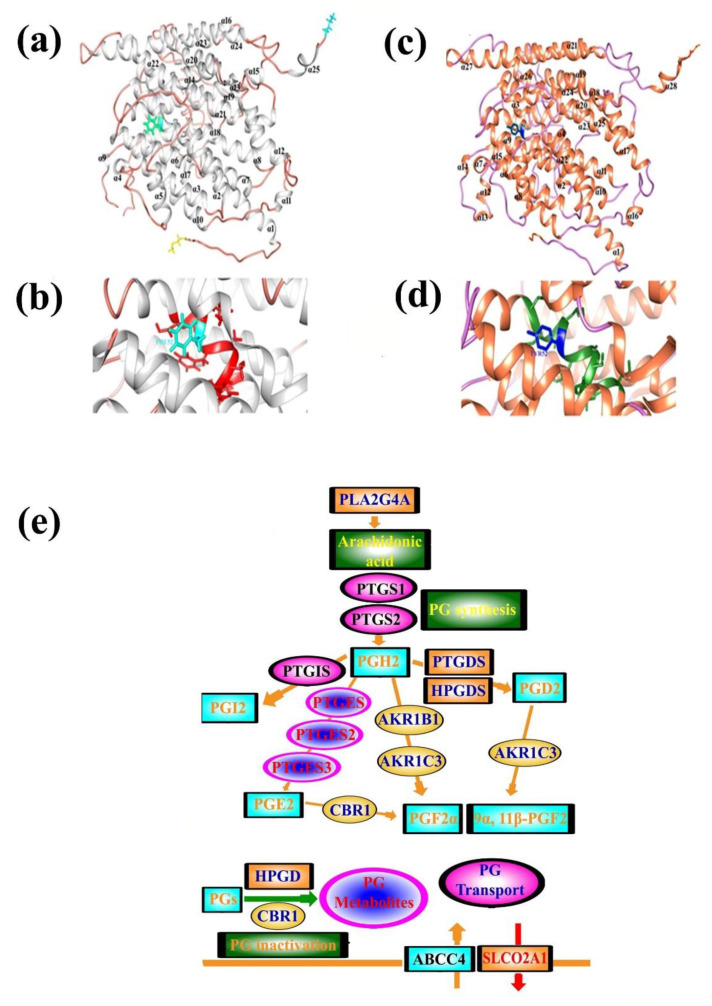
Protein structural and close-up view of SLCO2A1^WT^ and SLCO2A1^MUT^. (**a**) Structural representation of SLCO2A1^WT^ and (**c**) structural representation of SLCO2A1^PHE52TYR^ (**b**) Zoomed representation of SLCO2A1^WT^ and (**d**) zoomed representation of SLCO2A1^PHE52TYR^. PHE52 is depicted in cyan, while the TYR52 is presented in dark blue color. (**e**) Schematic representation of the pathway and key role of the SLCO2A1 and other key players in the transmembrane transporter. Different genes involved in the signaling and PGE2 biosynthesis pathway have been presented.

The novel SLCO2A1 variant (Phe52Tyr) was incorporated in the reference sequence of the SLCO2A1 to generate a homology-3D protein model of the protein. Gross level structural changes were observed in the secondary structural confirmation of the protein that might have unusual effect on the overall function of the protein. Multiple sequence alignment of both sequences revealed the substitution of PHE (F) instead of TYR (Y) 52, which is predicted to change the protein’s structure. SLCO2A1^WT^ was composed of 25 helices (Figure 3a), whereas in SLCO2A1^MUT^, the addition of 3α helices is predicted (Figure 3c). In TYR, we have the –OH group that is not present in PHE (Figure 3d), which might alter the bonding of side chains and results in a change in the protein structure (Figure 3a–d).

## 4. Discussion

Isolated digital/nail clubbing is a benign hereditary condition, which is a very rare; however, multiple associated conditions underlying etiology should be ruled out. Digital clubbing is associated with different types of infectious, neoplastic, inflammatory, and vascular diseases [17]. Generally, digital clubbing is roughly observed in 1% of all internal medicine admissions and profound disease association has been observed in 40% of those patients [18].

A Pakistani family was included in the current study affected by isolated nail clubbing. Clinico-genetic study revealed a novel biallelic novel sequence variant (c.155T>A; p.Phe52Tyr) in the *SLCO2A1* segregating within the family with the disease hallmarks. The nail phenotypes observed in the patients were consistent with ICNC phenotypes previously reported by Tariq et al. [8] and Shah et al. [19] in the Pakistani population. Tariq et al. [8], in a Pakistani family having isolated nail clubbing, reported a biallelic missense variant in the *HPGD* gene. Meanwhile, Shah et al. [19] reported a Pakistani family having features of isolated digital clubbing associated with *SLCO2A1*. Feature such as dermato-skeletal anomalies reported earlier [20,21], and incidence of anemia and hypoalbuminemia [2,20,22,23] were not observed in the affected individuals in the present study (Figure 1b). Genetic analysis of the family identified a novel substitution sequence variant (c.155T>A) in the *SLCO2A1* gene, the second homozygous sequence variant in *SLCO2A1* gene that is associated with ICNC phenotypes.

Homozygous and compound heterozygous variants within the HPGD have been determined as the underlying cause of PHO. HPGD encodes 15-hydroxyprostaglandin dehydrogenase, which is involved in the degradation of various substrates but more commonly catabolizes the prostaglandin (PG) E2 (PGE2), F2α (PGF2α), and B1 (PGB1). HPGD-carrying mutations have been associated with loss of enzymatic function. This is supported by urinary PGE2 being at an elevated level and decreased PGE-M levels, which is the excreted catabolic PGE2 end product. These findings suggest and correlate the PHO pathogenesis with the uncontrolled systemic PGE2 levels. PGs, including PGD2, PGE2, PGF2α, and PGI2, are active endocrinological lipids that play a significant role in a broad spectrum of biological processes such as inflammation, gastric secretion, airway homeostasis, vasoconstriction/-dilation, and bone formation as well as resorption. At physiologic pH, prostaglandins (PGs) traverse biologic membranes poorly. Accordingly, PG transport is carrier-mediated in many tissues, including the lung, choroid plexus, liver, anterior chamber of the eye, vagina, uterus, and placenta.

There are three possible roles for PGT. First, PGT might mediate the efflux of newly synthesized PGs from cells. Second, PGT might mediate epithelial PG transport. The third possible role of PGT is mediating PG clearance and degradation. Expression studies of a full-length human PGT cDNA clone in cultured cells showed that both rat and human PGT transported PGD2, as well as PGE1, PGE2, and PGF2a. As compared to rat PGT, human PGT showed a higher affinity for thromboxane-2. The best-characterized PG is the ubiquitous lipid mediator PGE2, which is synthesized from the precursor PGH2 by the prostaglandin synthases 1–3 (PTGES1-3). PGH2 synthesis from arachidonic acid is controlled by the PGH2 synthase PTGS1 and the PTGS2 (Figure 2e). PGE2 mediates its biological effects through the G protein-coupled prostaglandin E receptors 1–4 (encoded by PTGER1-4). PGE2 degradation to PGE-M is utilized in a first step by 15-PGDH to 15-oxo-PGE2, which is further degraded by PTGR1 and PTGR2 into PGE-M. Apart from synthesis and degradation, a critical step in PGE2 metabolism is the cellular release and uptake that is facilitated by organic anion transporters, such as SLCO2A1, SLCO4A1, and SLCO3A1 [2,7,8] (Figure 3e).

The gene *SLCO2A1* is located on chromosome 3q22.1-q22.2. The 14 coding exons of the *SLCO2A1* encode an organic anion-transporting polypeptide (NP_005621.2) (Figure 2a). SLCO2A1 is a protein of 643 residues with 12 transmembrane domains (Figure 2b). Protein 3D modeling of SLCO2A1 also revealed predominant structural alteration (Figure 3a–d) that predicts ablation in the characteristics of the protein at various level including folding, binding, shape, and function of the protein.

It has been observed that SLCO2A1 is expressed not only in the heart, lung, prostate, and skeletal muscle but also in other peripheral tissues including brain, testis, and ovary of several mammalian species, including humans [24,25]. Although the role of SLCO2A1 in prostaglandin (PG) metabolism still remains unclear, however, an important physiological function of PGT is the selective uptake of prostaglandins (Figure 3e) across the plasma membrane followed by its degradation inside the cell by 15-hydroxy-prostaglandin dehydrogenase, which occurs in the lung [26,27].

Thus, deficiency in HPGD and SLCO2A1 results in increased levels of prostaglandin E2 (PGE2), and in hypertrophic osteoarthropathy, primary, autosomal recessive 1 (PHOAR1; MIM 259100), and hypertrophic osteoarthropathy, primary, autosomal recessive 2 (PHOAR2; OMIM 614441), respectively [28], although there is overlap of clinical phenotypes between PHOAR1 and PHOAR2 in humans. The clinical spectrum of the affected members under study (homozygous *SLCO2A1* mutation) such as clubbing of distal phalanges, appeared early, while homozygous *HPGD* mutations also manifest the nail clubbing phenotype in early life. However, the degree of joint involvement, pachydermia, and arthritis in affected individuals having homozygous *SLCO2A1* mutations seems to be more pronounced than in individuals having *HPDG* mutations [19].

PHO disease pathophysiology is associated with loss of function (LOF) in the *SLCO2A1* and *HPGD* that results in increase in the PGE2 level in the extracellular compartment. PHO is the result of an excessive extracellular PGE2 level due to LOF variants in SLCO2A1 and HPGD. The concentration of PGE2 is regulated by PGT, which helps in the transportation of PGE2 and 15-HPGD from the extracellular toward the intracellular compartment. Similarly, their loss of function causes an increase in the PGE2 extracellular levels [29]. Thus, patients having variants in these genes have high urinary PGE2 levels [30].

In addition, PGE2 is also associated with stem cell differentiation into osteoblasts, which ultimately forms bones; thus PGD2 elevated levels causes bone pain and periostosis. Similarly, excessive PGE2 in the patients’ skin might be associated with cutis verticis gyrate, hyperhidrosis, seborrhea’ and pachydermia [29].

However, the *Slco2a1* knockout mice phenotypes were consistent with those observed in *Hpgd* knockouts [31]. *Slco2a1*^−/−^ mice were unable to survive, probably due to a closure defect of the ductus arteriosus [31]. In contrast to *Slco2a1*^−/−^ mice, individuals with *SLCO2A1* mutations did not reveal postnatal ductus arteriosus closure and had late onset of phenotypes after puberty, suggesting species specificities in PGE2 metabolism.

So far, only a heterozygous nonsense sequence variant ((c.754C>T, (p.R252*)) and a homozygous start loss sequence variant (c.1A>G, (p.M1V)) in the *SLCO2A1* gene have been associated with isolated nail clubbing [8,19]. Similarly, in a family with dominant nail clubbing, a heterozygous mutation (p.G104*) in the *SLCO2A1* gene was also reported, presenting features such as NSAID resistance and colon neoplasia [32]. Later, in a patient having sporadic ICNC, a heterozygous *SLCO2A1* nonsense mutation (p.R252*) was also reported [33]. Many pathogenic mutations in the *SLCO2A1* gene have been reported in patients suffering from PHOAD (OMIM 167100) and PHOAR2 (OMIM 614441), yet no clear genotype–phenotype relationship has been established. However, it has been observed that the phenotypic representation and symptoms of PHO were mild in PHOAD as compared to PHOAR2 patients, including less severe pachydermia and periostosis, and less frequent cutis verticis gyrata, acne, arthralgia, and anemia. In addition, the median urinary PGE2 level in PHOAD patients has been reported as less compared to that of PHOAR2 patients. Similarly, it has been observed that the degrees of arthritis, joint involvement, and pachydermia in patients with homozygous *SLCO2A1* variants are more pronounced as compared to those of patients having biallelic *HPDG* variants. This can be explicated by the presence of common second-site modifiers that strongly modulate the functional effects of the mutated SLCO2A1 protein and subsequent phenotypes.

The latest human genome mutation database version mentioned 101 mutations in the *SLCO2A1* gene, including 70 missense/nonsense, 14 splicing, 13 small deletions, 3 small insertions, and 1 small indel. In most cases, PHO was reported as a major clinical hallmark, whereas pachydermoperiostosis was the second most reported phenotype due to *SLCO2A1* mutations. In addition, some reported chronic enteropathy, and inflammatory bowel disease, whereas only two mutations have been reported causing isolated congenital nail clubbing.

HPGD homozygous or compound heterozygous mutations have been associated as a genetic cause of PHO, signifying the critical role of PGE2 in developing the disease associated manifestations [7,8]. This study has shown the development of PHO and digital clubbing due to PGE2 transporter encoding gene *SLCO2A1* mutations. PHO and digital clubbing due to *HPGD* and *SLCO2A1* mutations significantly pinpoint both PGT and 15-PGDH in controlling the termination of PGE2 signaling and subsequent oxidization into 15-oxo-PGE2, respectively [26].

Compared to the HPGD-associated phenotypic spectrum, late onset of disease-associated features has been observed in the patients carrying homozygous mutations of the *SLCO2A1* gene. Phenotype appearance begins with the clubbing of distal phalanges during puberty and pachydermia shortly after puberty. However, phenotypes such as arthritis, joint involvement, and pachydermia are more prominent in patients homozygous for *SLCO2A1* mutations than HPGD homozygous or compound heterozygous-affected individuals [7].

As POH is the most reported phenotype in patients with mutated *SLCO2A1*, an intra- and interfamilial variability in the phenotypic spectrum has also been observed correlating with time. Patients carrying homozygous *SLCO2A1* mutations develop clubbing of fingers and toes during or after puberty and hyperhidrosis of palms and soles. In addition, they are affected with periodic arthralgia, joint swelling, and bone deformity in early adulthood. In some patients within the same family, severe pachydermia with cutis gyrata, thickened eyelids, and seborrheic hyperplasia were seen after puberty. As for individuals with heterozygous *SLCO2A1* mutations, they either showed distal clubbing or no clinical abnormality, clearly suggesting a certain level of PGE2 uptake and metabolism that define the phenotypic consequence, or there might be the involvement of some factors regulating either PGE2 synthesis, degradation, or signaling that might influence the pathogenesis of PHO and distal clubbing [7]. Thus, a heterozygous carrier state for *SLCO2A1* mutations might result in isolated digital clubbing within adulthood in parallel with individuals heterozygous for *HPGD* mutations as a chronic rise in the PGE2 level also leads to late-onset clubbing [34]; thus, concluding the involvement of unknown genetic factors, environmental stimuli, and/or individual lifestyle may have some influence on the penetrance of isolated digital clubbing.

Similarly, a severe form of PHO phenotype has been observed in patients carrying *SLCO2A1* homozygous mutations, significantly overlapping with the PHO phenotype associated with HPGD mutations. Moreover, a milder phenotype characterized by isolated digital clubbing has been noticed in heterozygous *SLCO2A1* mutation patients. It has been shown that the PHO-associated *SLCO2A1* mutations close to the proximity of the PGE2 binding site and residue Glu78, Arg561, and Lys614 critical for PGE2 transport activity produce severe phenotypes as compared to the other mutations, thus suggesting disturbed PGE2 metabolism or PGE2 uptake directly [25,35]. The involvement of PGT in the cellular transport of PGE2 is a critical step within PG metabolism, and loss of PGT function is associated with a late onset human phenotype mainly affecting bone and skin tissue. Further investigation is required to fully delineate the molecular and cellular mechanisms contributing to the variable phenotypic expression of digital clubbing and PHO.

Genetic ectodermal dysplasias, such as rare syndromic or non-syndromic nail clubbing or associated disorders such as recessive and dominant hypertrophic osteoarthropathy, are heterogeneous and severe conditions. In such a scenario, proper genetic counseling, genetic testing (prenatal), and the introduction of new variants to newborn screening programs can significantly reduce the burden of such severe disorders [36]. The latest techniques, such as prenatal genetic testing for monogenetic disorders (PGT-M), can be used to make a proper molecular diagnosis. In addition, PGT and in vitro fertilization are available options for parents having syndromic conditions and still wishing to have future pregnancies [37,38,39,40]. Although there is no specific management for these cases, patients are examined frequently for any additional syndromic symptoms.

## 5. Conclusions

In summary, our findings confirm that *SLCO2A1* variants inactivate PGE2 transport, and cause isolated nail clubbing in humans. Further investigation is required to fully delineate the molecular and cellular mechanisms that contribute to the variable phenotypic expression.

## Figures and Tables

**Table 1:** Clinical/genetic features observed in the family under study carrying *SLCO2A1* mutation.

Phenotypic/Genotypic Characteristics	Family Members				
	VI-1	VI-2	VI-3	III-3	III-4
Sex	Male	Male	Female	Male	Female
Consanguinity	+	+	+	+	+
Clinical subtype	Complete	Complete	N.A	N.A	N.A
Mutation type	Homozygous Missense	Homozygous Missense	Homozygous Normal	Heterozygous carrier	Heterozygous carrier
SLCO2A1 allele 1	c.155T>A	c.155T>A	c.155T>T	c.155T>A	c.155T>A
SLCO2A1 allele 2	c.155T>A	c.155T>A	c.155T>T	c.155T>T	c.155T>T
Protein change	p.Phe52Tyr	p.Phe52Tyr	N.A	N.A	N.A
Digital clubbing	+	+	−	−	−

‘+’ presence of phenotype, ‘−’ absence of phenotype.

## Data Availability

Contact corresponding authors.
